# FGFR2 risk SNPs confer breast cancer risk by augmenting oestrogen responsiveness

**DOI:** 10.1093/carcin/bgw065

**Published:** 2016-05-28

**Authors:** Thomas M. Campbell, Mauro A.A. Castro, Ines de Santiago, Michael N.C. Fletcher, Silvia Halim, Radhika Prathalingam, Bruce A.J. Ponder, Kerstin B. Meyer

**Affiliations:** Department of Oncology, University of Cambridge, Cancer Research UK Cambridge Institute, Li Ka Shing Centre, Robinson Way, Cambridge CB2 0RE, UK and; ^1^Bioinformatics and Systems Biology Lab, Federal University of Paraná (UFPR), Polytechnic Center, Rua Alcides Vieira Arcoverde, 1225 Curitiba, Paraná 81520-260, Brazil; ^2^Present address: Deutsches Krebsforschungszentrum, Im Neuenheimer Feld 280, 69120 Heidelberg, Germany; ^3^Present address: Beatson Institute for Cancer Research, Switchback Road, Bearsden, Glasgow G61 1BD, UK; ^4^Present address: Abcam, Cambridge Science Park, Milton, Cambridge CB4 0FL, UK.

## Abstract

The fibroblast growth factor receptor 2 (FGFR2) locus is consistently the top hit in genome-wide association studies for oestrogen receptor-positive (ER^+^) breast cancer. Yet, its mode of action continues to be controversial. Here, we employ a systems biology approach to demonstrate that signalling via FGFR2 counteracts cell activation by oestrogen. In the presence of oestrogen, the oestrogen receptor (ESR1) regulon (set of ESR1 target genes) is in an active state. However, signalling by FGFR2 is able to reverse the activity of the ESR1 regulon. This effect is seen in multiple distinct FGFR2 signalling model systems, across multiple cells lines and is dependent on the presence of FGFR2. Increased oestrogen exposure has long been associated with an increased risk of breast cancer. We therefore hypothesized that risk variants should reduce FGFR2 expression and subsequent signalling. Indeed, transient transfection experiments assaying the three independent variants of the FGFR2 risk locus (rs2981578, rs35054928 and rs45631563) in their normal chromosomal context show that these single-nucleotide polymorphisms (SNPs) map to transcriptional silencer elements and that, compared with wild type, the risk alleles augment silencer activity. The presence of risk variants results in lower FGFR2 expression and increased oestrogen responsiveness. We thus propose a molecular mechanism by which FGFR2 can confer increased breast cancer risk that is consistent with oestrogen exposure as a major driver of breast cancer risk. Our findings may have implications for the clinical use of FGFR2 inhibitors.

## Summary

The fibroblast growth factor receptor 2 (FGFR2) locus is the ‘top hit’ in genome-wide association studies for breast cancer. Here, we examine the effect of FGFR2 signalling on transcriptional networks in breast cancer and propose a mechanism for FGFR2 risk single-nucleotide polymorphism function.

## Introduction

Breast cancer continues to be the most frequently diagnosed cancer globally and the leading cause of cancer death among females (1). The genetic factors contributing to breast cancer have been studied in some detail: rare genetic variants with high penetrance such as mutations in the *BRCA1/2* genes give rise to a 50–80% lifetime risk of developing breast cancer, but these rare mutations only account for ~5–7% of the total incidence of breast cancers (2). Genome-wide association studies have examined the common genetic variation in the population that is associated with breast cancer and have repeatedly identified fibroblast growth factor receptor 2 (FGFR2) as their ‘top hit’ (3–11), with the risk variants conferring increased risk for oestrogen receptor-positive (ER^+^) disease. Due to the common nature of the risk alleles at this locus, it is believed that the locus contributes to up to 16% of all breast cancers (4,12), suggesting a significant disease burden due to FGFR2.

The functional role of FGFR2 in the breast appears to depend on the cellular context and developmental stage. Experimental data obtained in mice show that FGFR2 is pro-proliferative in early mammary gland development, and reduced expression is associated with developmental defects in branching morphogenesis (13–16). In another study, low expression of FGFR2 was associated with lower numbers of breast tumour-initiating cells (17). However, in microdissected breast tumours FGFR2 mRNA and protein levels were reduced in tumour cells in comparison with paired normal breast epithelium, suggesting that tumourigenesis is associated with reduced FGFR2 expression (18). Thus, the molecular mechanism by which the FGFR2 protein contributes to tumourigenesis is not fully understood.

Genetic mapping studies of the *FGFR2* risk locus have led to the identification of three independent functional variants [independent, correlated highly associated variants] (19,20). Chromatin conformation studies have demonstrated that sequences around the risk single-nucleotide polymorphisms (SNPs) interact with the *FGFR2* promoter, making this the likely target gene. However, the effect of the risk variants on FGFR2 expression has remained controversial (20–23).

In this study, we examine the effect of FGFR2 activation on the transcriptional profiles of ER^+^ breast cancer cell lines. *In vitro*, FGFR2 can be activated by a number of different fibroblast growth factors (FGFs), although FGF10, a mesenchymal to epithelial signalling molecule, is the most potent agonist of FGFR2 and is the relevant FGFR2 ligand for breast epithelial cells (24,25). Here, we examine FGFR2 activation either by FGF10 stimulation or by small-molecule activators and consistently observe that FGFR2 signalling counteracts cell activation by oestrogen signalling. In keeping with this observation, we find that in transient transfection assays all three independent risk SNPs reduce transcriptional activation from the *FGFR2* promoter. Our findings suggest that reduced FGFR2 expression and signalling is associated with an increase in ER^+^ breast cancer risk.

## Materials and methods

### Cell culture

Michigan Cancer Foundation-7 (MCF-7) human breast cancer cells were cultured in Dulbecco’s modified Eagle’s medium (Invitrogen) supplemented with 10% fetal bovine serum (FBS) and antibiotics. ZR751, T47D and BT474 human breast cancer cells were cultured in RPMI (Invitrogen) supplemented with 10% FBS and antibiotics. SUM52PE human breast cancer cells were cultured in Ham/F-12 (Invitrogen) supplemented with 10% FBS, 5 µg/ml insulin, 1 µg/ml hydrocortisone and antibiotics. All cells were maintained at 37°C, 5% CO_2_. All cell lines were from the CRUK Cambridge Institute biorepository collection. Cell lines were authenticated by short tandem repeat genotyping using the GenePrint 10 (Promega) system and confirmed to be mycoplasma free. Cell line stocks were tested in September and October 2013, 2 months prior to microarray hybridization. Frozen aliquots were stored and after thawing not cultured for longer than 6 months.

### Stimulation of FGFR2 signalling

Cells were plated at 5×10^5^ cells/well in six-well dishes and left in complete medium overnight. Cell synchronization via oestrogen-starvation was then carried out for 3 days in oestrogen-free media (phenol red-free media supplemented with 5% charcoal dextran-treated FBS and 2mM l-glutamine), with media changes every 24h. Oestrogen-deprived cells were stimulated with 1nM β-estradiol (E2; Sigma) or 100ng/ml FGF10 (Invitrogen) in combination with 1nM E2, for 6 or 24h.

### RNA collection and microarray processing

Total RNA was extracted from cells using the miRNeasy Mini Kit (QIAGEN) and quality checked using an RNA 6000 Nano Chip on a 2100 Bioanalyser (Agilent). RNA (250ng; RNA integrity number > 9) was used for cRNA amplification and labelling using the Illumina TotalPrep-96 kit (Ambion). cRNA was hybridized to HumanHT-12 v4 Expression BeadChips according to the manufacturer’s protocol (Illumina WGGX DirectHyb Assay Guide 11286331 RevA). Bead level data were preprocessed to remove spatial artefacts, log2-transformed and quantile normalized using the beadarray package (26) from Bioconductor. The full microarray data sets have been deposited in Gene Expression Omnibus (GEO) under the SuperSeries number GSE74663.

### Quantitative RT-PCR

One microgram of total RNA was reverse transcribed using the High Capacity cDNA Reverse Transcription Kit (Applied Biosystems) and qRT-PCR performed using cDNA obtained from 10ng of total RNA. qRT-PCR was performed using an ABI 9800HT Sequence Detection System (Applied Biosystems) with SDS software version 2.3. All primers are listed in Supplementary Table 1, available at *Carcinogenesis* Online. Amplification and detection were carried out in 384-well Optical Reaction Plates (Applied Biosystems) with Power SYBR Green Fast 2x qRT-PCR Mastermix (Applied Biosystems). All expression data were normalized to DGUOK expression. Primer-specificity was confirmed at the end of each qRT-PCR run through the generation of single peaks in melt-curve analysis. Data analysis was performed using the 2^−ΔΔCT^ method (27). RNA from HMF3S cells was a kind gift from Dr M.Hoare at the CRUK Cambridge Institute.

### Western immunoblotting

Cells were grown in 10cm Petri dishes, washed in phosphate-buffered saline and lysed on ice in RIPA buffer with cOmplete Mini ethylenediaminetetraacetic acid-free protease inhibitor cocktail (Roche). Resulting cell lysates were passed through a fine-gauge syringe needle several times, centrifuged at 10000*g* for 1min and left at −80°C at least overnight. Protein samples were separated by sodium dodecyl sulphate–polyacrylamide gel electrophoresis using 4–12% Bis–Tris gels (Novex) for 2.5h (30min at 60V, 120min at 120V) and transferred by electrophoresis using an iBlot (Novex) for 7–8min onto a nitrocellulose membrane (iBlot Gel Transfer Stacks; Novex). Successful transfer of protein was confirmed using Ponceau S Solution (Sigma). Membranes were ‘blocked’ at room temperature for 1h with 5% (wt/vol) dried milk in Tris-buffered saline (TBS) with 0.1% Tween-20 (TTBS), washed 3× with TTBS and probed with the relevant primary antibody (anti-FGFR2, 1:200, Santa Cruz sc-122; anti-FGFR1, 1:1000, Santa Cruz sc-121; anti-ESR1, 1:5000, Santa Cruz sc-543 X; anti-β-actin, 1:5000, Cell Signalling) in blocking solution at 4°C overnight. Membranes were then rewashed with TTBS 3× and incubated with appropriate horseradish peroxidase-conjugated secondary antibody (1:10000, Amersham). Following further washing with TTBS, blots were treated with SuperSignal West Chemiluminescent Substrate (Thermo Scientific) and immunoreactive proteins detected by exposure to film (FUJIFILM). In all cases, loading controls of β-actin were run in parallel.

### Molecular cloning

The full-length *FGFR2* promoter was amplified from total genomic DNA from MCF-7 human breast cancer cells using primers 1 and 2 (Supplementary Table 2, available at *Carcinogenesis* Online). The PCR product was cloned into the pGL3-Basic Luciferase Reporter Vector (Promega), following XhoI/HindIII digestion, to generate a reporter construct in which the luciferase gene is transcribed from the *FGFR2* promoter. Reporter plasmids containing the putative *FGFR2* regulatory elements were generated by cloning ~800bp fragments, overlapping the *FGFR2* risk variants, upstream of the *FGFR2* promoter, following SacI/XhoI digestion. The 801bp regulatory element (RE1) containing the rs2981578 and rs35054928 SNPs was amplified from total genomic DNA from T47D (non-risk variants; T/-) and ZR751 (risk variants; C/C) human breast cancer cells using primers 3 and 4 (Supplementary Table 2, available at *Carcinogenesis* Online). Site-directed mutagenesis was performed on the ‘non-risk’ and ‘risk’ reporter plasmids using the QuikChange II XL Site-Directed Mutagenesis Kit (Agilent) to generate constructs with single SNPs (T/C and C/-). The 839bp regulatory element (RE2) containing the rs45631563 risk variant (A) was amplified from T47D genomic DNA using primers 5 and 6 (Supplementary Table 2, available at *Carcinogenesis* Online). Site-directed mutagenesis was performed on the ‘risk’ rs45631563 reporter plasmid using the QuikChange II XL Site-Directed Mutagenesis Kit (Agilent) to generate a construct containing the non-risk SNP variant of rs45631563 (T). The orientation and sequence of all cloned plasmids were confirmed by DNA sequencing (GATC Biotech).

### Luciferase reporter assay

MCF-7 cells were plated at 0.5×10^5^ cells/well in 24-well dishes and left in complete medium until 50–70% confluent. Cells were transfected with luciferase and β-galactosidase constructs at a concentration of 0.5 and 0.1 µg per well, respectively, using FuGENE HD Transfection Reagent (Promega), according to manufacturer’s protocol. After 24h, cells were lysed with Reporter Lysis Buffer (Promega) and luciferase and β-galactosidase assays were performed on a PHERAstar FS Microplate Reader (BMG LABTECH) using the appropriate assay kits (Promega), according to manufacturer’s protocol. Transfection of each reporter construct was performed in triplicate in each assay and a total of three assays were performed on three separate days.

### Transient transfection of siRNA

MCF-7 cells were transfected with ON-TARGETplus SMARTpool siRNA (Dharmacon) directed against *FGFR2* (L-003132-00), *ESR1* (L-003401-00) and a control non-targeting pool (D-001810-01) using Lipofectamine RNAiMax Reagent (Invitrogen), according to manufacturer’s protocol. Following addition of the transfection complexes, cells were incubated overnight before cell synchronization via oestrogen-starvation and stimulation of oestrogen/FGFR2 signalling was performed.

### Chromatin immunoprecipitation

For ESR1 ChIP-seq, cells were oestrogen starved for three consecutive days and E2-stimulated for 45min. ChIP-seq was performed as previously described (28). Briefly, cells were cross-linked in 1% formaldehyde for 10min. Nuclear extracts were prepared and sonicated using a Bioruptor (Diagenode) for 15min on the ‘high’ setting with cycles of 30s on and 30s off. Sonicated lysate was mixed with Protein A Dynabeads (Invitrogen) pre-incubated with antibody against ESR1 (Santa Cruz sc-543 X; 10 µg of antibody in 50 µl volume, diluted 1:25 in sonicated nuclear extract). Immunoprecipitated chromatin was used to prepare Solexa sequencing libraries. The full ChIP-seq data set has been deposited in GEO under the accession code GSE48930.

### Analysis of gene expression data

The Bioconductor package ‘limma’ (29) was used to call differentially expressed genes, and the log fold change metric was used to obtain the ranked phenotypes required for the gene set enrichment analysis (GSEA).

### Computation and validation of the ESR1 regulon

As previously described (30), we used mutual information in gene expression data [METABRIC data sets (31)] to calculate a regulatory network in which ESR1 is linked to all its potential target genes. We refer to these target genes as the ESR1 regulon. The network can be simplified or ‘filtered’ by applying the data processing inequality (dpi) function with increasingly stringent thresholds so that targets assigned to ESR1 are more likely to be direct targets. Validation of the regulon was carried out by testing whether ESR1 binding sites derived from ChIP-seq data in MCF-7 breast cancer cells are enriched near target genes. Distances between the nearest ESR1 binding site and the transcription start sites (TSS) were calculated and binned over a regular grid of 512 points by the ‘density’ function in ‘R’ to compute the kernel density estimates. Binding site density is given as the estimated number of sites in each point as a fraction of all TSS in the regulon. Statistical significance of the enrichment was assessed by comparison with random regulons or random genomic positions.

### Two-tailed GSEA

GSEA (32) assesses the skewed distribution of a selected gene set (*S*), here the ESR1 regulon, in a list of genes (*L*) ranked by a particular phenotype, in this case the gene expression response to stimulation by E2, E2 + FGF10 or si*ESR1* treatment. The two-tailed GSEA method is based on the Connectivity Map procedure (33). The regulon is split into two subgroups, positive targets (*A*) and negative targets (*B*), using Pearson’s correlation, whereas genes in the phenotype are ranked using the differentially expressed signatures (i.e. top-down phenotype). The distribution of *A* and *B* are then tested by the GSEA statistics in the ranked phenotype, producing independent enrichment scores (ES) for each subgroup. A good separation of the two distributions and maximum deviation from zero near opposite extremes is required for a clear association. Therefore, an additional step is executed testing the differential enrichment. The two-tailed GSEA was performed in ‘R’ using the function ‘tni.gsea2’ in the ‘RTN’ package (30,34) with 1000 permutations.

### Motif discovery

The HOMER motif discovery algorithm (findMotifsGenome.pl v4.7) was used to identify either known or novel transcription factor binding motifs (scripts to reproduce this analysis are provided as Supplementary Material, available at *Carcinogenesis* Online). We varied the dpi threshold to define the positive and negative targets within the ESR1 regulon. The derived gene lists are given in Supplementary Table 3, available at *Carcinogenesis* Online. The ‘R’ (v3.2.3) package TxDb.Hsapiens.UCSC.hg18.knownGenes (v3.2.2) was used to find ESR1 ChIP-seq binding peaks (GSE25710) within a symmetric 200 or 250kb window, centred on the gene TSS of the ESR1 regulon genes (hg18/NCBI36). These peaks were used as the search space for the motif finding algorithm. A more stringent dpi threshold or smaller window around the TSS generated similar results, albeit with less significant *P*-values.

### Additional information

Microarray data have been deposited in GEO under the SuperSeries number GSE74663 and the ‘R’ code that reproduces the results presented in this study is available in Bioconductor (http://bioconductor.org/packages/RTN/). The scripts used to reproduce the motif finding analysis are provided as Supplementary Material, available at *Carcinogenesis* Online. ChIP-seq data have been deposited in GEO under accession code GSE48930.

## Results

### FGFR2 signalling suppresses ESR1 signalling in ER^+^ breast cancer cells

Epidemiologic studies have shown that FGFR2 confers risk for ER^+^ disease only (3–11,35–37). We therefore employed oestrogen-dependent cell lines to examine the effect of FGFR2 activation using a systems biology approach.

Based on gene expression profiles in 2000 breast cancers, our previous network analysis (30) using ARACNe (38) defined a regulon (set of potential target genes) for ESR1. This algorithm infers target genes based on mutual information and tests for significance via permutation and bootstrap analysis. The inferred targets can be direct or indirect. To remove indirect targets, we applied a dpi step in which increasingly stringent thresholds were set. We then asked whether the genes in the more stringently defined regulon display greater ESR1 binding, as assayed by ESR1 ChIP-seq. There was a significant enrichment when the ESR1 regulon was compared with random regulons or to random genomic sites (Supplementary Figure 1, available at *Carcinogenesis* Online). ESR1 binding density increased when the more stringently defined regulons were tested, and all the subsequent analysis used this filtered regulon.

**Figure 1.**
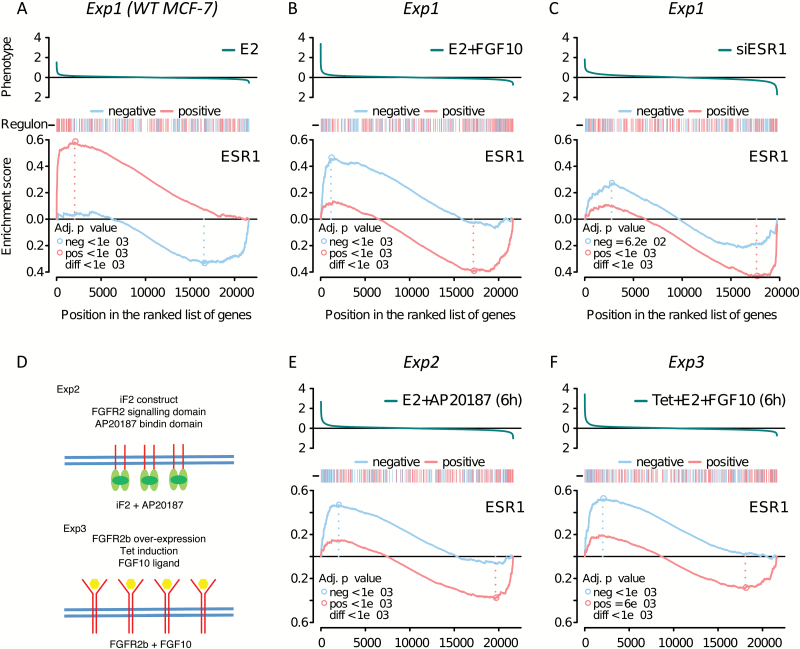
Response of the ESR1 regulon in MCF-7 cells to oestrogen and FGFR2 signalling. The top panel in each plot shows the response of all genes on the microarray, with the *y*-axis representing the fold change in gene expression to any given stimulus (phenotype), as shown. The bar beneath the phenotype shows red marks for activated and blue marks for repressed members of the ESR1 regulon. The GSEA plots show the running enrichment score for positive (red line) and negative (blue line) targets in the ESR1 regulon in MCF-7 cells. The *x*-axis applies to all three panels and lists all genes ranked by the phenotype. (**A–C**) MCF-7 cells were stimulated with (A) 1nM E2 for 6h, (B) 1nM E2 plus 100ng/ml FGF10 for 6h and (C) 50nM siRNA directed against *ESR1* for 24h. (**D**) Cartoon of two additional FGFR2 signalling model systems. MCF-7 cells stably overexpressing the FGFR2 kinase domain, which can be activated by a small-molecule crosslinker (AP20187) (Exp2) and MCF-7 cells overexpressing full-length FGFR2b from a Tet-inducible promoter (Exp3) are depicted. (**D** and **E**) GSEA plots after cell stimulation for Exp2 (D) and Exp3 (E), after 6h.

We first characterized the behaviour of the ESR1 regulon in MCF-7 cells. We examined the effect of oestrogen stimulation and compared the resultant gene expression profile with that of unstimulated cells. ‘Limma’ analysis revealed a similar number of activated and repressed genes. In an analysis of the ESR1 regulon as a whole, the opposing effects of these two gene sets may obscure each other. We therefore split the regulon into a group of activated and a group of repressed genes, based on the Pearson’s correlation of gene expression between ESR1 and each target in tumours (31). A negative value indicates a repressed target gene and a positive value indicates an activated target gene. We then asked how the two sets are distributed in a ranked list of oestrogen-responsive genes using two-tailed GSEA. The two-tailed GSEA tests whether positive or negative targets for a transcription factor of interest are enriched at each extreme of a particular response (e.g. oestrogen stimulation). As expected, GSEA revealed that oestrogen stimulation upregulates genes that are positively regulated by ESR1 in tumours (Figure 1A). Conversely, genes that are negatively regulated by ESR1 are enriched among the genes that are repressed on oestrogen stimulation, again entirely in keeping with expectations. We next examined how the ESR1 regulon responds to FGF10 stimulation (on a background of oestrogen signalling). Compared with the oestrogen treatment alone, the oestrogen plus FGF10 treatment leads to a downregulation of ESR1 positive targets, whereas ESR1-negative target genes are upregulated (Figure 1B). This strongly suggests that FGFR2 signalling counteracts ESR1 signalling in these cells. To confirm this, we transfected siRNA against *ESR1* into MCF-7 cells and tested the effect on the gene expression profiles. The direction of the response was the same as that for FGF10 signalling (Figure 1C), in keeping with the notion that FGF10 inhibits ESR1 signalling.

In addition to the induction described above (Exp1), we have previously established two additional model systems to examine FGFR2 signalling in MCF-7 cells (30): cells overexpressing the FGFR2 kinase domain, which can be activated by a small-molecule crosslinker (AP20187; Exp2), and cells overexpressing full-length FGFR2b from a Tet-inducible promoter (Exp3). In all three of these systems, we find that FGFR2 signalling represses the ESR1 regulon (Figure 1D–F), with cells overexpressing FGFR2 showing a stronger response after 24h (Supplementary Figure 2, available at *Carcinogenesis* Online). Our results demonstrate that the effect is likely to be mediated by FGFR2 and not through other receptors potentially activated by FGF10.

**Figure 2.**
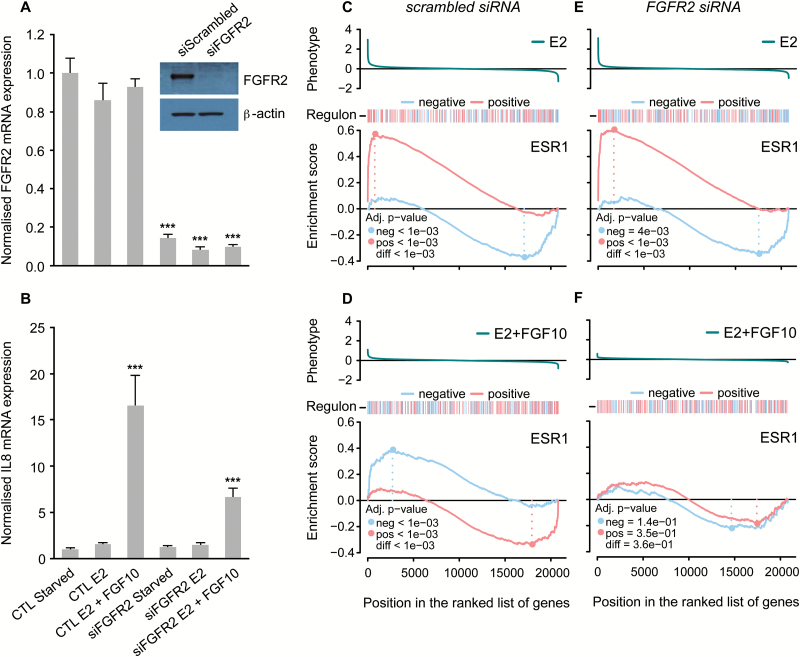
Knocking down FGFR2 dampens ESR1 regulon repression in MCF-7 cells. Relative mRNA expression of FGFR2 (**A**) and IL8 (**B**) in MCF-7 cells following cell synchronization and stimulation of oestrogen/FGF10 signalling [oestrogen-starvation for three consecutive days followed by a further 6h oestrogen-starvation (starved) or treatment with either 1nM E2 (E2) or 1nM E2 plus 100ng/ml FGF10 (E2 + FGF10) for 6 h]. CTL: control condition; si*FGFR2*: cells transfected with siRNA against *FGFR2* for a period of 24h prior to cell synchronization and stimulation of oestrogen/FGF10 signalling. All data were normalized to DGUOK expression [*n* = 10, two separate experiments, *P* < 0.001 (***), ns (not significant), one-way ANOVA and SNK correction, error bars = SEM]. Inset: representative western immunoblots showing expression of FGFR2 and β-actin proteins in MCF-7 cells following transfection with scrambled siRNA (siScrambled) or siRNA directed against *FGFR2* (si*FGFR2*), and cell synchronization (*n* = 3 for both blots). (**C–F**) GSEA plots showing the degree of enrichment for positive and negative targets in the ESR1 regulon in MCF-7 cells that have either been transfected with a scrambled siRNA sequence (C and D) or siRNA directed against *FGFR2* (E and F), following treatment with 1nM E2 for 6h (C and E) and 1nM E2 plus 100ng/ml FGF10 for 6h (D and F).

To confirm that FGFR2 is required in this response, we next examined the effect of reducing FGFR2 expression on the oestrogen response. We transfected MCF-7 cells with siRNA against *FGFR2* before cell synchronization and subsequent stimulation of FGFR2 signalling (on a background of oestrogen signalling). Knock-down of FGFR2 reduced FGFR2 mRNA and protein levels in si*FGFR2*-transfected versus untransfected cells (Figure 2A). *IL8* is one of the genes most strongly induced upon FGFR2 stimulation and we demonstrated that FGF10-induced IL8 mRNA expression is reduced in si*FGFR2*-transfected cells (Figure 2B). Microarray analysis of gene expression followed by GSEA was carried out as described for Exp1–3 above. While knocking down FGFR2 had no effect on the ESR1 regulon response to oestrogen treatment (Figure 2C and E), the FGFR2 knock-down significantly dampened the response of the ESR1 regulon to FGF10 treatment (Figure 2D and F). In the untransfected cells, FGF10 reversed the response to oestrogen and genes that are activated by oestrogen (shown in a red line in Figure 2C and E) are now repressed (Figure 2D). After si*FGFR2* transfection, this reversal is much less pronounced (Figure 2F). This suggests that the negative effects of FGF10 stimulation on oestrogen signalling in MCF-7 cells are mediated via FGFR2. This effect was not detected in a simple analysis of differential gene expression. Our results highlight the power of a network-based analysis to dissect complex transcriptional responses.

**Figure 3.**
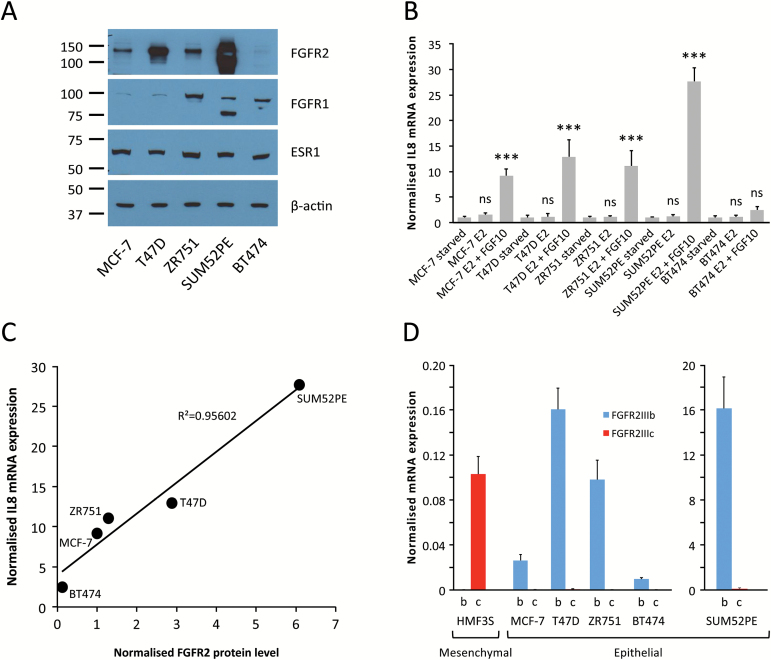
FGFR2 expression and activity in ER^+^ breast cancer cells. (**A**) Representative western immunoblots showing expression of FGFR2, FGFR1, ESR1 and β-actin proteins in ER^+^ human breast cancer cell lines (*n* = 3 for all blots). (**B**) Relative mRNA expression of IL8 in five different ER^+^ human breast cancer cell lines (MCF-7, T47D, ZR751, SUM52PE and BT474) following cell synchronization and stimulation of oestrogen/FGF10 signalling [oestrogen-starvation for three consecutive days followed by a further 6h oestrogen-starvation (starved) or treatment with either 1nM E2 (E2) or 1nM E2 plus 100ng/ml FGF10 (E2 + FGF10) for 6 h]. All data were normalized to DGUOK expression [*n* = 10, two separate experiments, *P* < 0.001 (***), ns (not significant), one-way ANOVA and SNK correction, error bars = SEM]. (**C**) Correlation plot showing the relationship between FGFR2 protein level [determined by densitometry analysis of the FGFR2 western immunoblots shown in (A)] and IL8 mRNA expression following FGF10 stimulation [shown in (B)] for the five ER^+^ human breast cancer cell lines. (**D**) Relative mRNA expression of the FGFR2IIIb (b) and FGFR2IIIc (c) isoforms in the five ER^+^ human breast cancer cell lines. HMF3S, human mammary fibroblast cell line used as a positive control for the FGFR2IIIc primers. HMF3S is a mesenchymal cell line, all others are epithelial. Expression levels in SUM52PE cells are shown on a different scale because the FGFR2 locus is amplified in this cell line. All data were normalized to DGUOK expression (*n* = 10, two separate experiments, error bars = SEM).

To ensure that the effects are not specific to MCF-7 cells, we expanded our analysis of gene expression changes after FGFR2 signalling to include additional ER^+^ cell lines: MCF-7, T47D, ZR751, SUM52PE and BT474. First, we assessed the levels of FGFR2 protein expression in these cell lines and found that expression levels varied greatly (Figure 3A): the lowest expression was found in BT474 cells which have hardly detectable levels of FGFR2, with higher expression seen in MCF-7, ZR751 and T47D cells. Highest expression was in SUM52PE cells, which carry an amplification of the *FGFR2* locus (39). Following oestrogen-starvation, all cell lines were stimulated with oestrogen and oestrogen plus FGF10. Assaying IL8 mRNA by qRT-PCR indicated that the response to FGF10 was proportional to FGFR2 expression (Figure 3B and C). FGF10 is the most potent agonist of FGFR2 and is the relevant FGFR2 ligand for breast epithelial cells (24,25). It is specific for the FGFR2IIIb isoform, and qRT-PCR confirmed that only the FGFR2IIIb isoform is expressed in ER^+^ epithelial breast cancer cells (Figure 3D). Next, we carried out a microarray analysis of gene expression in response to FGF10 treatment. Of note, in BT474 cells only a very small number of genes responded to FGF10 stimulation. Previous work suggested that FGF10 can also interact weakly with the FGFR1 receptor (40,41), which is expressed in BT474 cells (Figure 3A). However, the very small response indicates that the effect is negligible in these cells and that FGF10 signals predominantly through FGFR2. The unresponsiveness of BT474 cells is visualized in Supplementary Figure 3, available at *Carcinogenesis* Online, showing that there is only a small number of shared differentially expressed genes between BT474 and the other ER^+^ breast cancer cell lines in a genome-wide analysis. BT474 cells can therefore serve as a negative control in our experiments. In contrast, the other four cell lines displayed a large number of concordant changes in gene expression after FGFR2 activation (Supplementary Figure 3, available at *Carcinogenesis* Online).

**Figure 4.**
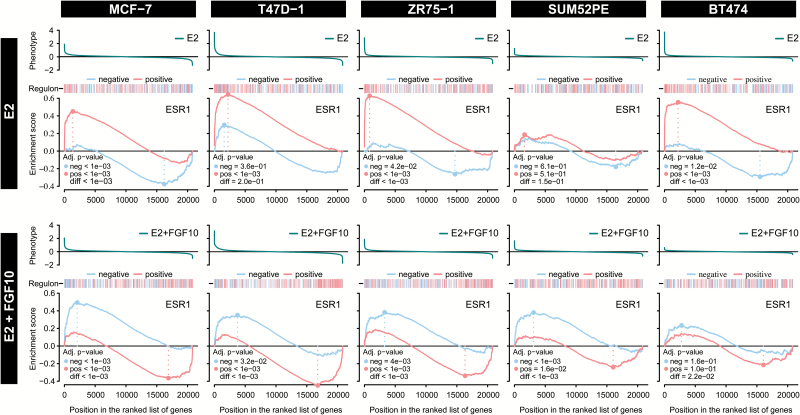
Effect of E2 and FGF10 on the ESR1 regulon is consistent across ER^+^ cell lines. GSEA plots showing the degree of enrichment for positive and negative targets in the ESR1 regulon in five different ER^+^ human breast cancer cell lines (MCF-7, T47D, ZR751, SUM52PE and BT474) following treatment with either 1nM E2 (E2) or 1nM E2 plus 100ng/ml FGF10 (E2 + FGF10) for 6h.

Two-tailed GSEA was carried out for the ESR1 regulon after the two different treatments (oestrogen alone and oestrogen plus FGF10) in the five ER^+^ cell lines. For T47D and ZR751 cells, results are very similar to the response seen in MCF-7s (Figure 4). In contrast, BT474 cells, which have low FGFR2 expression, display a very low enrichment score for FGF10-repressed genes in the ESR1 regulon. Furthermore, there is a lower oestrogen response in SUM52PE cells, which express high levels of FGFR2. Presumably, the high levels of FGFR2 expression lead to some constitutive FGFR2 signalling, even in the absence of FGF10 ligand. These results provide further evidence that FGFR2 signalling counteracts cell activation by oestrogen signalling.

### Motif analysis of oestrogen-responsive genes

As yet, we do not understand the molecular mechanism by which FGFR2 exerts its effect on gene expression. We asked whether distinct regulatory elements could be detected at the ESR1 binding sites of activated and repressed genes. Using the HOMER motif discovery algorithm, we found that very similar motifs were identified near ESR1 binding sites at oestrogen-activated and oestrogen-repressed genes. As expected, these included the oestrogen response element itself as well as FOXA1, AP2 and FOS/JUN binding sites. The *P*-value for enrichment for the oestrogen response element motif was somewhat higher in positively regulated genes, but otherwise no consistent differences were found when varying the motif finding parameters (Supplementary Table 4, available at *Carcinogenesis* Online). Similar motifs were also identified when searching for *de novo* motifs (data not shown).

### Risk SNPs reduce FGFR2 expression within a silencer context

**Figure 5.**
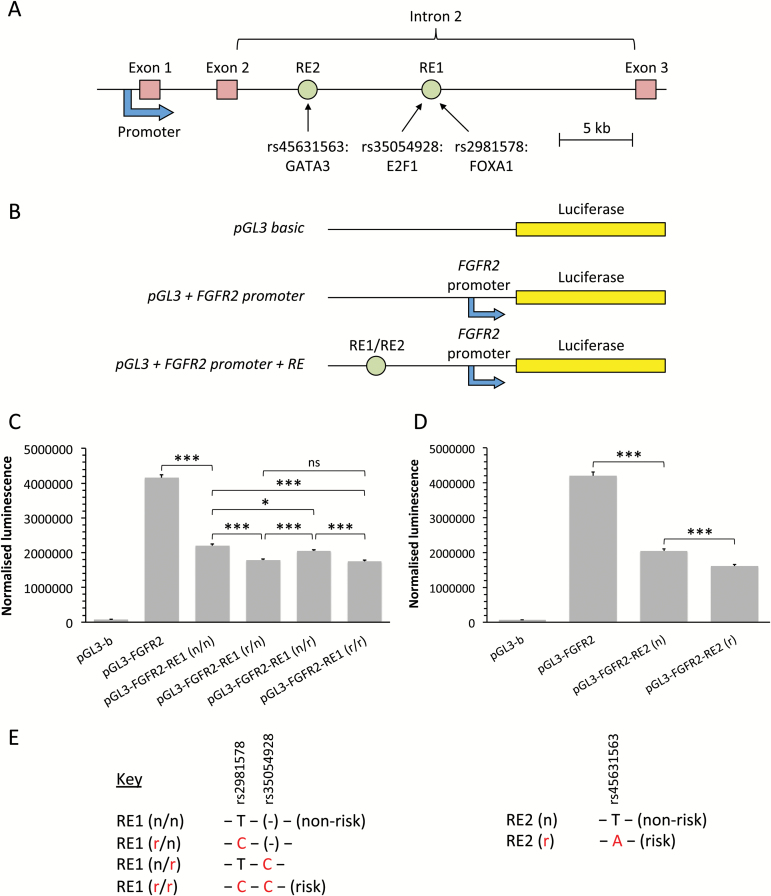
Risk SNPs reduce FGFR2 expression within a silencer context. (**A**) Schematic depiction of the start of the *FGFR2* gene, showing the location of the cloned *FGFR2* promoter and two response elements (RE1 and RE2) containing the three SNPs in intron 2. (**B**) Schematic depiction of the reporter constructs used in the luciferase reporter assays. The full-length *FGFR2* promoter was cloned into the multiple cloning site of a pGL3-Basic Luciferase Reporter Vector in order to generate a reporter construct in which the luciferase gene is transcribed from the *FGFR2* promoter. The putative *FGFR2* regulatory elements (RE1 and RE2) containing the risk SNP variants were then cloned upstream of the *FGFR2* promoter. (**C** and **D**) Luciferase luminescence in MCF-7 cells 24h post-transfection of reporter constructs, normalized to β-galactosidase expression. pGL3-b: pGL3-Basic vector; pGL3-FGFR2: pGL3-Basic vector + *FGFR2* promoter; pGL3-FGFR2-RE1/2: pGL3-Basic vector + *FGFR2* promoter + regulatory element 1/2; n: non-risk SNP variant; r: risk SNP variant [*n* = 9, three separate experiments, *P* < 0.05 (*), *P* < 0.001 (***), ns (not significant), one-way ANOVA and SNK correction, error bars = SEM]. (**E**) Key showing which risk SNP variants are present in the regulatory element of each reporter construct.

Exposure to oestrogen has long been known to be one of the key risk factors for breast cancer development, and anti-oestrogen therapies are a fundamental current strategy for breast cancer prevention and treatment (42–44). As FGFR2 reduces the potency of oestrogen activation, we hypothesized that the risk variants in the *FGFR2* locus should reduce transcriptional activation. rs2981578, rs35054928 and rs45631563 have previously been identified as the most likely causative variants in each of the three independent, correlated highly associated variants defined through genetic mapping of the *FGFR2* risk locus (20). To assess the role of these risk SNPs (Supplementary Table 5, available at *Carcinogenesis* Online) on FGFR2 expression, we first generated a luciferase reporter construct in which the luciferase gene is transcribed from the *FGFR2* promoter. Upstream of this we cloned the two putative *FGFR2* regulatory elements, RE1 and RE2 (Supplementary Figure 4, available at *Carcinogenesis* Online), and tested the resultant constructs in transient transfection assays in MCF-7 cells. We found that both RE1 (chr10: 123339661 to 123340461; 801bp) and RE2 (chr10: 123349160 to 123349998; 839bp) function as silencer elements, reducing transcription from the *FGFR2* promoter 1.9- and 2.0-fold, respectively (Figure 5). In keeping with the proposed silencer function, we note that the transcriptional repressors YY1, SIN3A and HDAC2 (Supplementary Figure 4, available at *Carcinogenesis* Online) bind within RE1. In the presence of the two risk variants at rs2981578 and rs35054928 in RE1, transcription is further reduced by 21%. Similarly, in the presence of the single risk variant at rs45631563 in RE2, transcription is 22% lower compared with the RE2 construct containing the non-risk allele. Thus, the risk variants of all three SNPs increase silencer activity of the regulatory elements.

## Discussion

**Figure 6.**
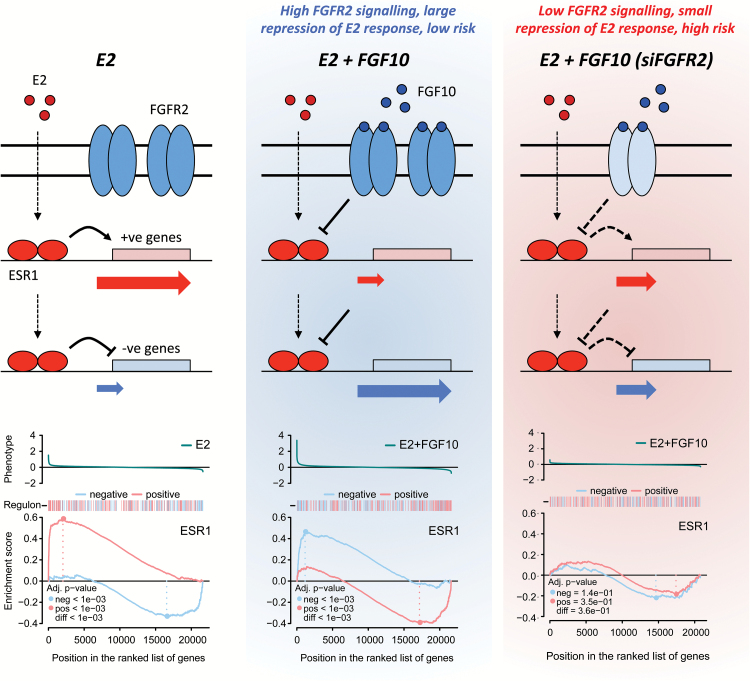
The relationship between FGFR2 signalling and the oestrogen response in ER^+^ breast cancer cells. Diagram showing how the data presented in this study supports the hypothesis that FGFR2 signalling has an inhibitory effect on oestrogen signalling. Presented are three GSEA plots showing the response of the ESR1 regulon to E2 treatment, E2 plus FGF10 treatment and knock-down of ESR1 with siRNA. Above each GSEA plot is a schematic representing how we propose the positively and negatively regulated genes in the ESR1 regulon respond to the various cell treatments. Reduced FGFR2 expression as a result of the presence of risk SNPs in the second intron of the *FGFR2* gene would result in an increase in the oestrogen response in this context.

Here, we demonstrate that in breast cancer cell lines FGFR2 signalling is able to oppose the effect of oestrogen signalling (Figure 6). This repression occurs independent of the mechanism of FGFR2 activation, either via its ligand, FGF10, or via dimerization of the FGFR2 kinase domain. Furthermore, we find that the response is proportional to the level of FGFR2 expression, with higher expression leading to a stronger induction of known response genes such as *IL8* and more pronounced inhibition of the oestrogen response (in SUM52PE cells). Conversely, depletion of FGFR2 via siRNA treatment abrogates the suppressive effect of FGF10 on the oestrogen regulon. As oestrogen is the key driver of ER^+^ breast cancer, our results suggest that an increase in disease risk should be associated with reduced FGFR2 expression.

Consistent with this we find that all three risk variants within the *FGFR2* locus act to reduce transcriptional activation at the *FGFR2* promoter. The effect of rs2981578 was significantly stronger than that of rs35054928. These results are in keeping with the findings (20) that in MCF-7 cells there is an 8.5-fold increase in FOXA1 binding at the risk allele of rs2981578, whereas the difference in nuclear protein (E2F1) binding between the two alleles at rs35054928 was only 2.4- to 3.8-fold. Due to genetic linkage, the two risk SNPs are co-inherited, conferring reduced FGFR2 expression. Our previous analysis of a multimerized rs2981578 site had suggested that the disease-associated allele increases *FGFR2* transcription (21). However, in these earlier experiments, the multimerized rs2981578 site was not tested in its normal chromosomal context as part of a larger regulatory element. We now demonstrate that this element functions as a silencer element. Therefore, these apparently contradictory findings can easily be reconciled. In line with our earlier studies, we propose that FOXA1 binds more strongly to the risk allele of rs2981578, increasing chromatin accessibility (20) and allowing access to transcriptional repressors such as YY1, SIN3A and HDAC2, leading to a reduction in *FGFR2* promoter activity. The third risk variant, rs45631563, is relatively infrequent (3%), thus making a smaller contribution to overall risk, but also repressed *FGFR2* transcription.

The observation that risk variants reduce FGFR2 expression is consistent with a study that assayed purified, cultured mammary epithelial cells and reported that the presence of the rs2981578 risk allele led to lower FGFR2 expression (23). Another study reported lower levels of FGFR2 mRNA and protein expression in tumour compared with normal breast tissue (18). This study examined a small number of patients (21 matched tumour-normal pairs and 10 tumour samples without corresponding normal tissue), but samples were microdissected to maximize any detectable differences. In contrast, a recent eQTL study of normal breast tissue failed to see an association between the risk alleles and FGFR2 expression (45), but in this study, tissue was not microdissected. It is not clear how this might fit with experimental data that suggest FGFR2 is important in increased branching during mammary gland development and in the generation of breast tumour-initiating cells (13–17): it is likely that FGFR2 may play distinct roles at different stages of breast development.

Our findings of a link between FGFR2 signalling and oestrogen responsiveness are also supported by epidemiologic studies (46), which have reported an interaction between hormone therapy and FGFR2 genotype. The SNP identified in this study was independent from the SNPs most strongly associated with breast cancer risk in genetic fine mapping studies (20), for which a hormone interaction could only be detected in some cohorts (46–48).

Most receptor tyrosine kinases are thought to be able to act as oncogenes if deregulated. Indeed, *FGFR1* is frequently amplified or mutated in prostate adenocarcinoma (23%), lung squamous carcinoma (23%) and breast cancer (14.3%) (TCGA), and FGFR inhibitors are being trialled clinically. Yet our results suggest that FGFR2 plays a suppressive role in breast cancer. In support of this, genomic studies found that *FGFR2* amplifications (1.8%) or mutations (1.3 %) are relatively rare in breast cancer (cBioportal, TCGA). The results we present here suggest that FGFR inhibitors may augment the risk of breast cancer development and this should be borne in mind when selecting patient groups for treatment with pan-FGFR inhibitors.

In conclusion, our analysis of FGFR2 signalling and the effect of breast cancer risk variants suggest that the role of *FGFR2* germline variants in breast cancer risk may be linked to reducing a cell’s ability to respond to oestrogen activation. The role of FGFR2 in breast cancer risk may therefore be distinct from the role normally associated with FGFRs in tumour progression. The molecular mechanism we propose establishes a link between the inherited risk for breast cancer conferred by germline variation in *FGFR2* and environmental effects (oestrogen exposure).

## Supplementary material

Supplementary Tables 1–5 and Figures 1–4 can be found at http://carcin.oxfordjournals.org/

## Funding

Breast Cancer Research Foundation (BCRF); Cancer Research UK (CRUK grant reference: C20/A17031).

## Author contributions

T.M.C. carried out the luciferase experiments and cell stimulation experiments. M.A.A.C. computed the GSEA and validation analysis of the filtered regulons. I.S. examined *FGFR2* gene expression in tumour data and the correlation of gene expression in cell lines. S.H. processed the raw microarray gene expression data. M.N.C.F. generated ChIP-seq data and performed the motif analysis. R.P. assisted in cloning luciferase constructs. K.B.M. and B.A.J.P. proposed the idea and obtained funding for this work. T.M.C., K.B.M., M.A.A.C. and B.A.J.P. designed the experiments and wrote the manuscript. All authors have read and approved the final manuscript.

*Conflict of Interest Statement:* None declared.

## Supplementary Material

Supplementary Data

## Abbreviations

ER: oestrogen receptor
FBS: fetal bovine serum
FGFR: fibroblast growth factor receptor
GSEA: gene set enrichment analysis
SNP: single-nucleotide polymorphism
TBS: Tris-buffered saline
TSS: transcription start sites

## References

[1] Jemal A. (2011) Global cancer statistics. CA Cancer J. Clin., 61, 69–90. 21296855 10.3322/caac.20107

[2] Roy R. (2012) BRCA1 and BRCA2: different roles in a common pathway of genome protection. Nat. Rev. Cancer, 12, 68–78. 22193408 10.1038/nrc3181 PMC4972490

[3] Easton D.F. (2007) Genome-wide association study identifies novel breast cancer susceptibility loci. Nature, 447, 1087–1093. 17529967 10.1038/nature05887 PMC2714974

[4] Hunter D.J. (2007) A genome-wide association study identifies alleles in FGFR2 associated with risk of sporadic postmenopausal breast cancer. Nat. Genet., 39, 870–874. 17529973 10.1038/ng2075 PMC3493132

[5] Garcia-Closas M. (2008) Heterogeneity of breast cancer associations with five susceptibility loci by clinical and pathological characteristics. PLoS Genet., 4, e1000054. 18437204 10.1371/journal.pgen.1000054 PMC2291027

[6] Liang J. (2008) Genetic variants in fibroblast growth factor receptor 2 (FGFR2) contribute to susceptibility of breast cancer in Chinese women. Carcinogenesis, 29, 2341–2346. 18845558 10.1093/carcin/bgn235

[7] Udler M.S. (2009) FGFR2 variants and breast cancer risk: fine-scale mapping using African American studies and analysis of chromatin conformation. Hum. Mol. Genet., 18, 1692–1703. 19223389 10.1093/hmg/ddp078 PMC2733817

[8] Barnholtz-Sloan J.S. (2010) FGFR2 and other loci identified in genome-wide association studies are associated with breast cancer in African-American and younger women. Carcinogenesis, 31, 1417–1423. 20554749 10.1093/carcin/bgq128 PMC2950798

[9] Orr N. (2011) Genetic variants at chromosomes 2q35, 5p12, 6q25.1, 10q26.13, and 16q12.1 influence the risk of breast cancer in men. PLoS Genet., 7, e1002290. 21949660 10.1371/journal.pgen.1002290 PMC3174231

[10] Michailidou K. (2013) Large-scale genotyping identifies 41 new loci associated with breast cancer risk. Nat. Genet., 45, 353–361, 361e1–2. 23535729 10.1038/ng.2563 PMC3771688

[11] Salehi Z. (2015) Evaluation of FGFR2 gene polymorphism in women with breast cancer. Cell. Mol. Biol. (Noisy-le-grand), 61, 94–97. 26025410

[12] Raskin L. (2008) FGFR2 is a breast cancer susceptibility gene in Jewish and Arab Israeli populations. Cancer Epidemiol. Biomark. Prev., 17, 1060–1065. 10.1158/1055-9965.EPI-08-0018 PMC6172344 18483326

[13] Dillon C. (2004) A crucial role for fibroblast growth factor signaling in embryonic mammary gland development. J. Mammary Gland Biol. Neoplasia, 9, 207–215. 15300014 10.1023/B:JOMG.0000037163.56461.1e

[14] Parsa S. (2008) Terminal end bud maintenance in mammary gland is dependent upon FGFR2b signaling. Dev. Biol., 317, 121–131. 18381212 10.1016/j.ydbio.2008.02.014

[15] Cerliani J.P. (2012) Associated expressions of FGFR-2 and FGFR-3: from mouse mammary gland physiology to human breast cancer. Breast Cancer Res. Treat., 133, 997–1008. 22124578 10.1007/s10549-011-1883-6 PMC7511987

[16] Pond A.C. (2013) Fibroblast growth factor receptor signaling is essential for normal mammary gland development and stem cell function. Stem Cells, 31, 178–189. 23097355 10.1002/stem.1266 PMC3690809

[17] Kim S. (2013) FGFR2 promotes breast tumorigenicity through maintenance of breast tumor-initiating cells. PLoS One, 8, e51671. 23300950 10.1371/journal.pone.0051671 PMC3534701

[18] Zhu X. (2010) Genetic and epigenetic mechanisms down-regulate FGF receptor 2 to induce melanoma-associated antigen A in breast cancer. Am. J. Pathol., 176, 2333–2343. 20348248 10.2353/ajpath.2010.091049 PMC2861098

[19] Edwards S.L. (2013) Beyond GWASs: illuminating the dark road from association to function. Am. J. Hum. Genet., 93, 779–797. 24210251 10.1016/j.ajhg.2013.10.012 PMC3824120

[20] Meyer K.B. (2013) Fine-scale mapping of the FGFR2 breast cancer risk locus: putative functional variants differentially bind FOXA1 and E2F1. Am. J. Hum. Genet., 93, 1046–1060. 24290378 10.1016/j.ajhg.2013.10.026 PMC3852923

[21] Meyer K.B. (2008) Allele-specific up-regulation of FGFR2 increases susceptibility to breast cancer. PLoS Biol., 6, e108. 18462018 10.1371/journal.pbio.0060108 PMC2365982

[22] Sun C. (2010) rs2981582 is associated with FGFR2 expression in normal breast. Cancer Genet. Cytogenet., 197, 193–194. 20193855 10.1016/j.cancergencyto.2009.11.006 PMC2831800

[23] Huijts P.E. (2011) Allele-specific regulation of FGFR2 expression is cell type-dependent and may increase breast cancer risk through a paracrine stimulus involving FGF10. Breast Cancer Res., 13, R72. 21767389 10.1186/bcr2917 PMC3236336

[24] Itoh N. (2014) Fgf10: a paracrine-signaling molecule in development, disease, and regenerative medicine. Curr. Mol. Med., 14, 504–509. 24730525 10.2174/1566524014666140414204829

[25] Zhang X. (2014) FGF ligands of the postnatal mammary stroma regulate distinct aspects of epithelial morphogenesis. Development, 141, 3352–3362. 25078648 10.1242/dev.106732 PMC4199126

[26] Dunning M.J. (2007) beadarray: R classes and methods for Illumina bead-based data. Bioinformatics, 23, 2183–2184. 17586828 10.1093/bioinformatics/btm311

[27] Livak K.J. (2001) Analysis of relative gene expression data using real-time quantitative PCR and the 2(-Delta Delta C(T)) Method. Methods, 25, 402–408. 11846609 10.1006/meth.2001.1262

[28] Schmidt D. (2009) ChIP-seq: using high-throughput sequencing to discover protein-DNA interactions. Methods, 48, 240–248. 19275939 10.1016/j.ymeth.2009.03.001 PMC4052679

[29] Smyth G.K (2004) Linear models and empirical bayes methods for assessing differential expression in microarray experiments. Stat. Appl. Genet. Mol. Biol., 3, Article3. 16646809 10.2202/1544-6115.1027

[30] Fletcher M.N. (2013) Master regulators of FGFR2 signalling and breast cancer risk. Nat. Commun., 4, 2464. 24043118 10.1038/ncomms3464 PMC3778544

[31] Curtis C. (2012) The genomic and transcriptomic architecture of 2,000 breast tumours reveals novel subgroups. Nature, 486, 346–352. 22522925 10.1038/nature10983 PMC3440846

[32] Subramanian A. (2005) Gene set enrichment analysis: a knowledge-based approach for interpreting genome-wide expression profiles. Proc. Natl Acad. Sci. USA, 102, 15545–15550. 16199517 10.1073/pnas.0506580102 PMC1239896

[33] Lamb J. (2006) The Connectivity Map: using gene-expression signatures to connect small molecules, genes, and disease. Science, 313, 1929–1935. 17008526 10.1126/science.1132939

[34] Castro M.A.A. (2014) RTN: reconstruction of transcriptional networks and analysis of master regulators. R package. http://bioconductor.org/packages/release/bioc/html/RTN.html (14 October 2015, date last accessed).

[35] Cen Y.L. (2013) Associations of polymorphisms in the genes of FGFR2, FGF1, and RBFOX2 with breast cancer risk by estrogen/progesterone receptor status. Mol. Carcinog., 52 (suppl. 1), E52–E59. 23143756 10.1002/mc.21979

[36] O’Brien K.M. (2014) Breast cancer subtypes and previously established genetic risk factors: a Bayesian approach. Cancer Epidemiol. Biomark. Prev., 23, 84–97. 10.1158/1055-9965.EPI-13-0463 PMC3947131 24177593

[37] Slattery M.L. (2013) Associations with growth factor genes (FGF1, FGF2, PDGFB, FGFR2, NRG2, EGF, ERBB2) with breast cancer risk and survival: the Breast Cancer Health Disparities Study. Breast Cancer Res. Treat., 140, 587–601. 23912956 10.1007/s10549-013-2644-5 PMC3860319

[38] Basso K. (2005) Reverse engineering of regulatory networks in human B cells. Nat. Genet., 37, 382–390. 15778709 10.1038/ng1532

[39] Tannheimer S.L. (2000) Characterization of fibroblast growth factor receptor 2 overexpression in the human breast cancer cell line SUM-52PE. Breast Cancer Res., 2, 311–320. 11056689 10.1186/bcr73 PMC13919

[40] Zhang X. (2006) Receptor specificity of the fibroblast growth factor family. The complete mammalian FGF family. J. Biol. Chem., 281, 15694–15700. 16597617 10.1074/jbc.M601252200 PMC2080618

[41] Cui Y. (2013) Expression and functions of fibroblast growth factor 10 in the mouse mammary gland. Int. J. Mol. Sci., 14, 4094–4105. 23434672 10.3390/ijms14024094 PMC3588087

[42] Advani P. (2014) Current strategies for the prevention of breast cancer. Breast Cancer (Dove Med. Press), 6, 59–71. 24833917 10.2147/BCTT.S39114 PMC4018310

[43] Ban K.A. (2014) Epidemiology of breast cancer. Surg. Oncol. Clin. N. Am., 23, 409–422. 24882341 10.1016/j.soc.2014.03.011

[44] Clemons M. (2001) Estrogen and the risk of breast cancer. N. Engl. J. Med., 344, 276–285. 11172156 10.1056/NEJM200101253440407

[45] Seo J.H. (2013) Deconvoluting complex tissues for expression quantitative trait locus-based analyses. Philos. Trans. R. Soc. Lond. B. Biol. Sci., 368, 20120363. 23650637 10.1098/rstb.2012.0363 PMC3682728

[46] Prentice R.L. (2009) Variation in the FGFR2 gene and the effects of postmenopausal hormone therapy on invasive breast cancer. Cancer Epidemiol. Biomark. Prev., 18, 3079–3085. 10.1158/1055-9965.EPI-09-0611 PMC2783392 19861516

[47] Andersen S.W. (2013) Breast cancer susceptibility associated with rs1219648 (fibroblast growth factor receptor 2) and postmenopausal hormone therapy use in a population-based United States study. Menopause, 20, 354–358. 23435034 10.1097/GME.0b013e318268ca46 PMC3549049

[48] Rebbeck T.R. (2009) Hormone-dependent effects of FGFR2 and MAP3K1 in breast cancer susceptibility in a population-based sample of post-menopausal African-American and European-American women. Carcinogenesis, 30, 269–274. 19028704 10.1093/carcin/bgn247 PMC2722148

