# Supplementary material for: FGFR2 risk SNPs confer breast cancer risk by augmenting oestrogen responsiveness
Source: Carcinogenesis. 2016 May 28;37(8):741–50. doi: 10.1093/carcin/bgw065 (PMC4967216; doi:10.1093/carcin/bgw065)
Supplement: Supplementary Data [file supp_37_8_741__index.html]

FGFR2 risk SNPs confer breast cancer risk by augmenting oestrogen responsiveness — FGFR2 risk SNPs confer breast cancer risk by augmenting oestrogen responsiveness — FGFR2 risk SNPs confer breast cancer risk by augmenting oestrogen responsiveness — Supplementary Data 

# FGFR2 risk SNPs confer breast cancer risk by augmenting oestrogen responsiveness

## Supplementary Data

Data files

- Supplementary Data - Supplementary Data
- Supplementary Data - Supplementary Data
- Supplementary Data - Supplementary Data
