# Supplementary material for: FGFR2 risk SNPs confer breast cancer risk by augmenting oestrogen responsiveness
Source: Carcinogenesis. 2016 May 28;37(8):741–50. doi: 10.1093/carcin/bgw065 (PMC4967216; doi:10.1093/carcin/bgw065)
Supplement: Supplementary Data [file supp_bgw065_Supplementary_Material_10Mar.pdf]

Supplementary Material for

FGFR2 risk SNPs confer breast cancer risk by augmenting estrogen responsiveness

Thomas M. Campbell[1], Mauro A. A. Castro[2], Ines de Santiago[1], Michael N. C. Fletcher[1†], Silvia Halim[1†], Radhika Prathalingam[1†], Bruce A. J. Ponder[1] and Kerstin B. Meyer[1*]

Supplementary Figure legends

Supplementary Figures

Supplementary Tables

Supplementary Figure legends

Supplementary Figure 1. Validation of the ESR1 regulon. Plot showing the ESR1 binding density obtained from ChIP-seq data at the transcription start sites (TSS) of the predicted members of the ESR1 regulon (red), random regulons (green) and random genetic sites (blue). The regulon for ESR1 was calculated using an increasing threshold in the dpi analysis (A, least stringent (1), through to D (0.05, 0.01 and 0), most stringent). The more stringent the threshold in the dpi is, the stronger the link with the ESR1 binding data (*i.e.* the ChIP-seq signal increases with predicted weight of the TF-interactions).

Supplementary Figure 2. GSEA plots showing the degree of enrichment for positive and negative targets in the ESR1 regulon in MCF-7 cells following treatment with 1 nM E2 using the three different FGFR2 signalling systems (Exp1-3) described in Figure 1, at a time-point of 24 hours.

Supplementary Figure 3. Differential gene expression after FGFR2 signalling across five ER^+ breast cancer cells. Pairwise correlation of log2 fold-changes between shared differentially expressed genes (FDR<0.01) across five different ER^+ human breast cancer cell lines (MCF-7, T47D, ZR751, SUM52PE and BT474) following stimulation with FGF10, against a backdrop of E2 stimulation (E2 only versus E2 plus FGF10). BT474 shares only few differentially regulated genes with the other four cell lines. Correlation results are based on the Pearson correlation coefficient. Individual genes are shown as grey spots, with overlapping spots resulting in darker shading.

Supplementary Figure 4. Screenshots from the UCSC Genome Browser showing the regions cloned from genomic DNA for the two putative response elements (RE1 and RE2) and for the *FGFR2* promoter, showing associated DHSs, SNPs, TF binding regions and HMM chromatin states. SNPs rs2981578, rs35054928 and rs45631563 are highlighted within red boxes.

Supplementary Figure 1

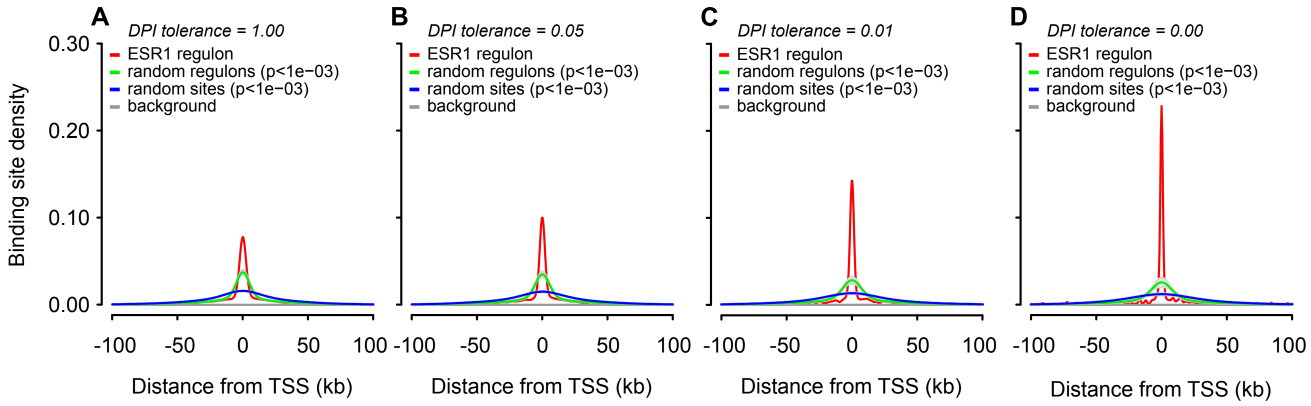

Supplementary Figure 2

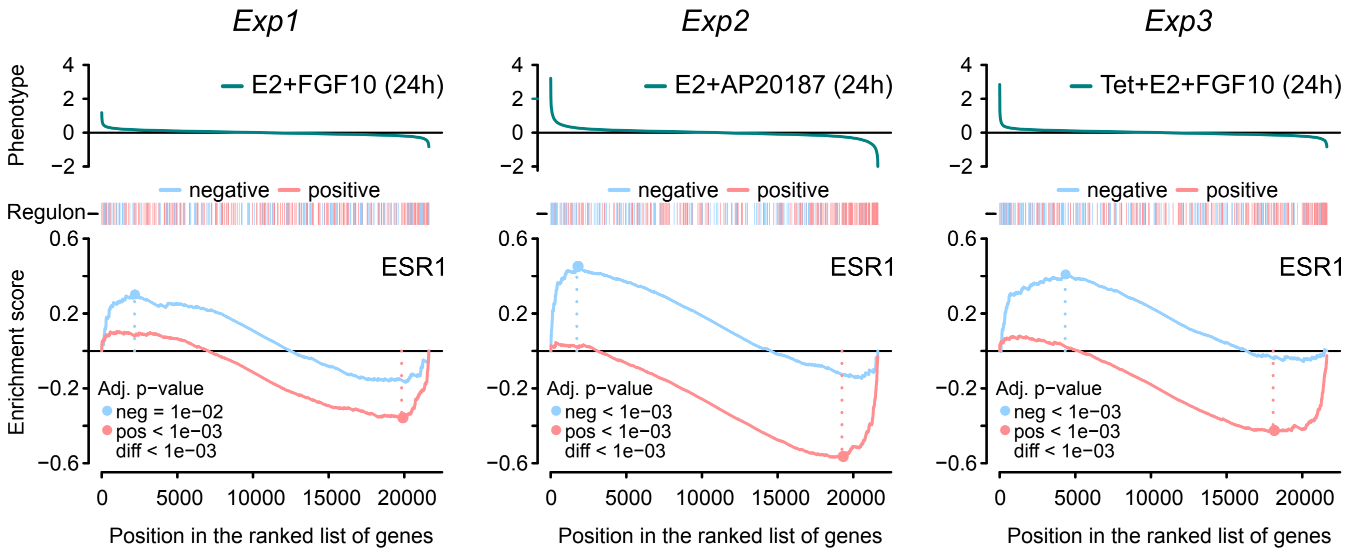

Supplementary Figure 3

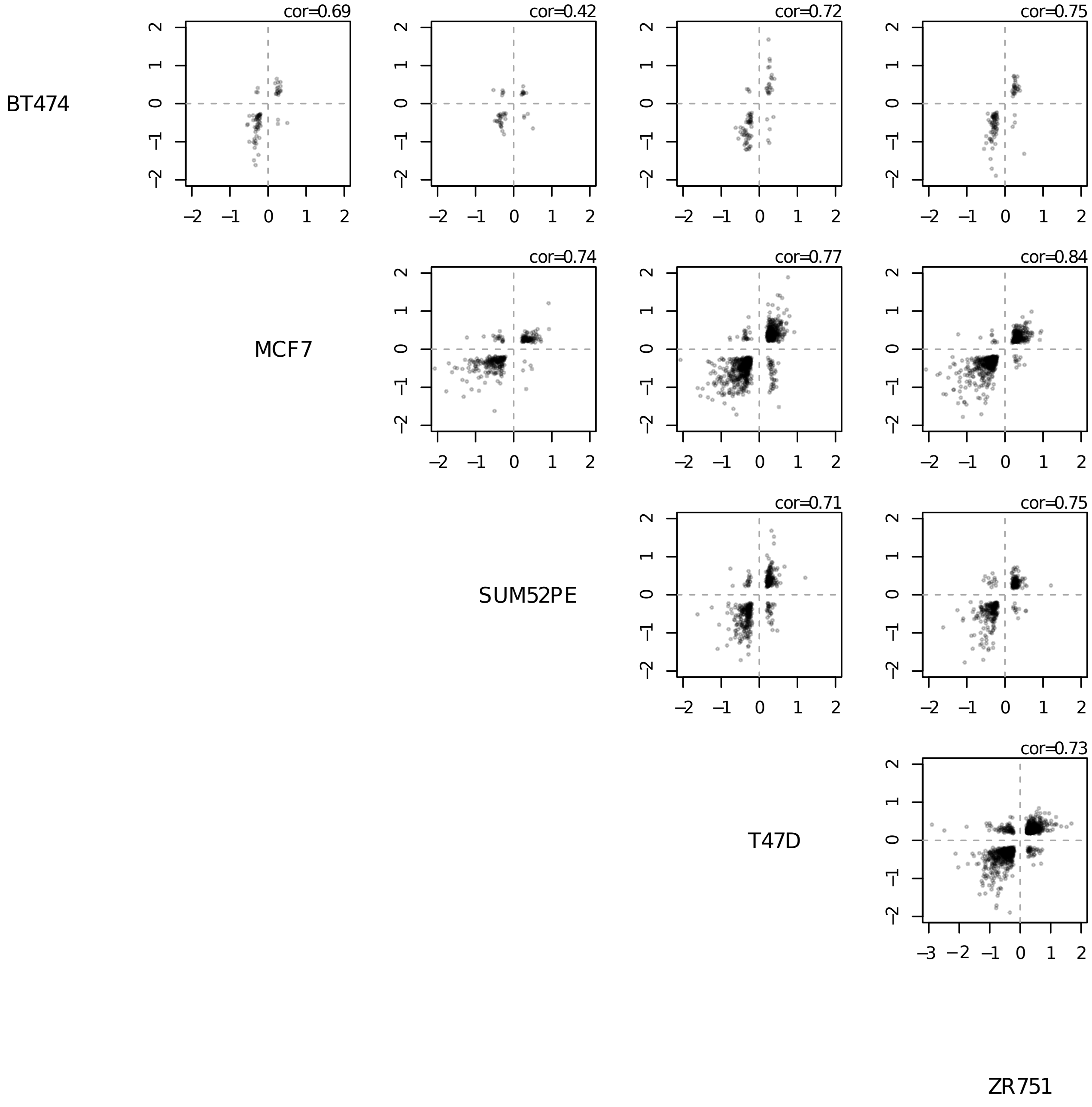

Supplementary Figure 4

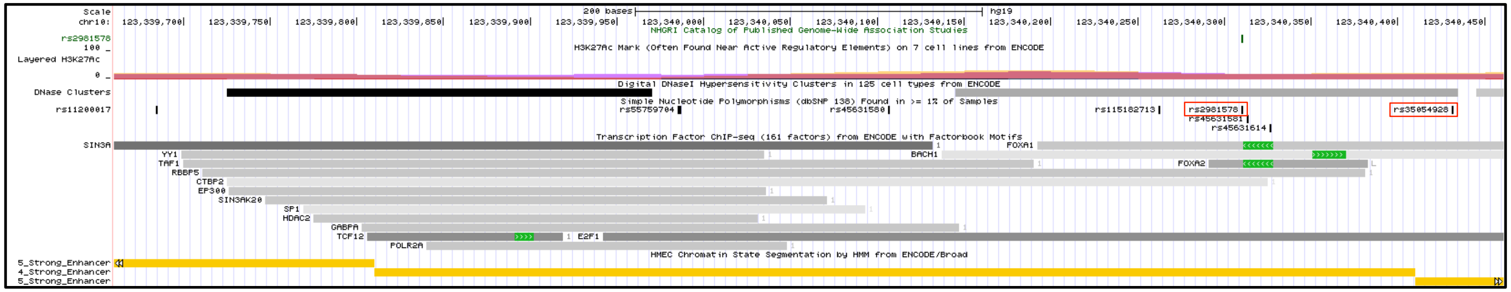

RE1: 801 bp

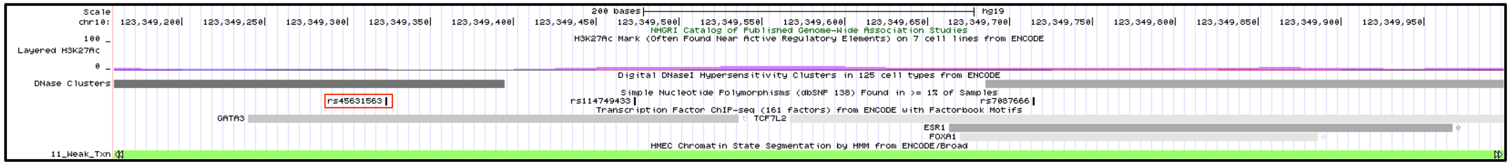

RE2: 839 bp

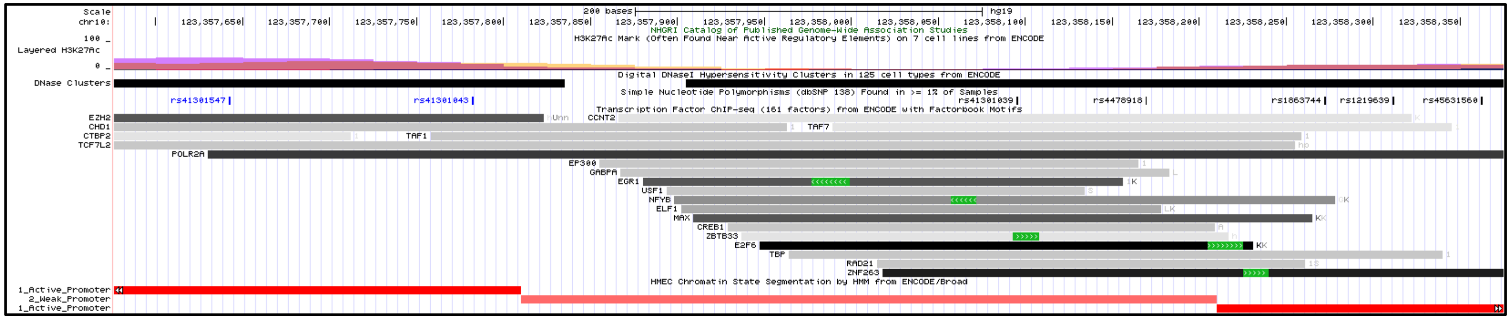

FGFR2 promoter: 799 bp

Supplementary Tables

Supplementary Table 1. Primers used in qRT-PCR to determine mRNA expression in human breast cancer cell lines.

Gene	***Forward primer (5'-3')***	***Reverse primer (5'-3')***
DGUOK	GCTGGTGTTGGATGTCAATG	GCCTGAACTTCATGGTATTGG
IL8	AAAGCTTTCTGATGGAAGAGAG	CCAGGAATCTTGTATTGCATC
FGFR2	GTCAGTGAGAACAGTAACAACAAG	GTAGCCTCCAATGCGATGC
FGFR2IIIb	GATAAATAGTTCCAATGCAGAAGTGCT	TGCCCTATATAATTGGAGACCTTACA
FGFR2IIIc	AGATTGAGGTTCTCTATATTCGGAATG	TTCTCTTCCAGGCGCTGG

Supplementary Table 2. Cloning primers used to amplify the *FGFR2* promoter and regulatory elements surrounding *FGFR2* risk SNPs from genomic DNA.

Region	***Forward primer (5'-3')***	***Reverse primer (5'-3')***
FGFR2 promoter	Primer 1: CGCAAGCTTACGCAGAAGAGTGGTCCTTG	Primer 2: CGCCTCGAGAGTATCAAGCAGGCGCATTT
RE1	Primer 3: GCGCTCGAGGTGGCCAAGGCTACAAAAGA	Primer 4: GCGGAGCTCTCCCAGAAAGCCTACATTCG
RE2	Primer 5: GCGCTCGAGGCTTGCCAGATTTCTGCTCT	Primer 6: GCGGAGCTCTTATAGGGCACCAGCAGAGG

RE1, regulatory element 1 (containing rs2981578 and rs35054928 variants)
RE2, regulatory element 2 (containing rs45631563 variant)

Supplementary Table 3. The ESR1 regulon. Table showing the positive and negative targets in the ESR1 regulon with varying dpi thresholds.

(ATTACHED FILE: ESR1 regulon.cvs)

Supplementary Table 4. Motif analysis using the HOMER motif discovery algorithm. HOMER was used to identify known TF binding motifs at ESR1 ChIP-seq peaks (GSE25710) for both sets of genes in the dpi-filtered ESR1 regulon; those positively regulated by estrogen stimulation and those negatively regulated by estrogen stimulation. The table shows the ranked list of the top motifs identified by the algorithm using a dpi threshold of 0.05 and a search area of either 250 or 200 kb from the TSS in each gene list.

	Positively regulated genes in ESR1 regulon			***Negatively regulated genes in ESR1 regulon***		
	Search window around TSS	***250 kb***	***200 kb***	*Search window around TSS*	***250 kb***	***200 kb***
	Number of ChIP-seq peaks	***1941***	***1678***	*Number of ChIP-seq peaks*	***1503***	***1220***
TF binding motif	ERE 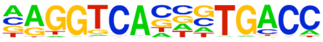	$1e^{-263}$	$1e^{-215}$	ERE 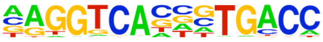	$1e^{-175}$	$1e^{-135}$
	FOXA1 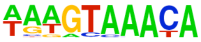	$1e^{-101}$	$1e^{-89}$	FOXA1 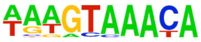	$1e^{-74}$	$1e^{-59}$
	FOXA2 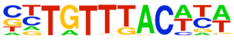	$1e^{-77}$	$1e^{-68}$	AP2α 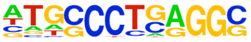	$1e^{-66}$	$1e^{-52}$
	AP2α 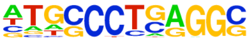	$1e^{-60}$	$1e^{-54}$	AP2γ 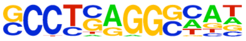	$1e^{-62}$	$1e^{-45}$
	ERRβ 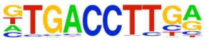	$1e^{-60}$	$1e^{-51}$	FOXA2 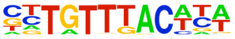	$1e^{-55}$	$1e^{-46}$
	FOSL2 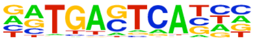	$1e^{-59}$	$1e^{-50}$	ERRβ 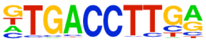	$1e^{-48}$	$1e^{-39}$
	AP2γ 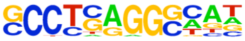	$1e^{-56}$	$1e^{-55}$	FOSL2 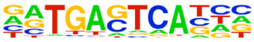	$1e^{-45}$	$1e^{-35}$

Supplementary Table 5. Putative functional SNPs within the second intron of *FGFR2.*

SNP (position on Chr10)	***Alleles (non-risk/risk)***	***RAF***	***DHS***	***Known TF Binding Site***
rs2981578 (123340311)	T/C	0.50	Yes	FOXA1*
rs35054928 (123340431)	-/C	0.44	Yes	E2F1*
rs45631563 (123349324)	T/A	0.97	Yes	GATA3†

Abbreviations are as follows: SNP, single nucleotide polymorphism; RAF, risk allele frequency; DHS, DNase I hypersensitive site; TF, transcription factor.

*Taken from the UCSC Genome Browser and from functional data [20]

†Taken from the UCSC Genome Browser only

Scripts

Scripts used with the HOMER motif discovery algorithm to identify both known and novel transcription factor binding motifs at ESR1 ChIP-seq binding peaks.

(ATTACHED FILE: run_motif_analysis_esr1peaks_hg18.sh)
